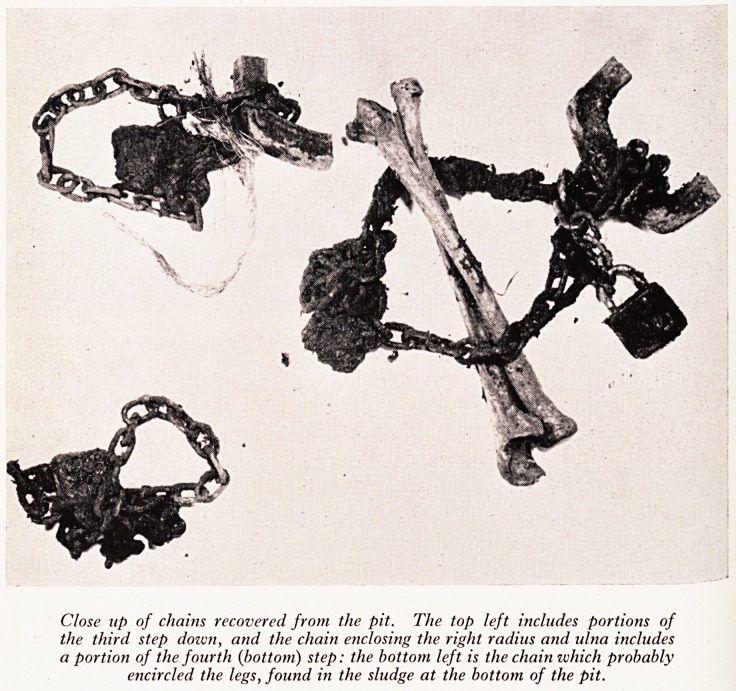# The Skeleton in the Pit

**Published:** 1961-04

**Authors:** G. Stewart Smith

**Affiliations:** Area Pathologist, Exeter and Devon Clinical Area


### THE SKELETON IN THE PIT

BY

G. STEWART SMITH, M.A., M.D.

*Area Pathologist, Exeter and Devon Clinical Area*

On 2nd January 1960 a farmer, walking near an overgrown naval camp whch had last been used in 1946, decided, in view of recent flooding, to lift the cover of an inspection pit leading from an old sewage tank belonging to the camp. At the bottom of the pit he saw a human skull, and immediately replaced the cover and informed the police. I was told of this finding, and next morning I visited the camp site with Chief-Superintendent Langman of the Devon County Constabulary; it was a very desolate spot and seldom visited (Plate XV).

The pit, which was covered by a heavy concrete slab measuring 3 ft. x 2 ft., was 7 ft. 2 in. deep and 3 ft. 5 in. wide at the bottom. There were four metal loop steps arranged in zig-zag fashion with 18 in. between each step: the third step was 3 ft. 7 in. from the bottom of the pit (about the level of the neck of a man kneeling on the ground).

### MEDICO LEGAL REPORT

The report given to the Coroner on the findings in the pit was as follows:

On the third step there was a chain secured by a padlock and turned twice over the step, and on the inner surface of the loop formed by this chain there was the second vertebra of the neck and a blue garment, the collar of which was also adherent to the chain.

The fourth step had two chains, secured by padlocks, as shown in the illustrations. These formed three loops, and in one of these loops there were the right radius and ulna (Plates XVI and XVII).

Along the bottom of the pit lay a gulley completely filled with thick sludge, and in this there was another chain, secured by padlock, which had two loops which could well, from their size and position, have been round the ankles.

The body had been reduced to the condition of a completely disarticulated skeleton. The skull was on the floor of the pit, and the lower jaw was lying apart from it. The position of the leg bones indicated that they had been stretched out along the floor of the pit with the feet near the end away from the steps.

All the large bones of the body were identified and several of them, mentioned later in this report, were measured. There were 16 vertebrae (out of a possible 24), 20 ribs (out of a possible 24) and 14 small bones from the hands and feet (out of a possible 106). One patella was identified.

The bones all had well-marked muscle ridges of male type: they had not lost any appreciable amount of weight.

The following bones were measured and from Pearson's formula an estimate was made of the stature of the deceased:

| *Bone* | *Length* | *Estimated stature based on this measurement* |
|---|---|---|
| Rt. Femur | 48·9 cm | 68 in. |
| Lt. Femur | 48·3 cm. | |
| Rt. Humerus | 35·7 cm | 68·37 in. |
| Lt. Humerus | 35·5 cm | |
| Rt. Tibia | 40·3 cm | 68 in. |
| Lt. Tibia | 40·5 cm | |
| Rt. Radius | 25·6 cm | 68·5 in. |
| Lt. Radius | 25·7 cm | |

The skull was of moderate size, and from the condition of the suture lines and the angle of the jaw, was that of a young man. The length of the skull was $7\frac{1}{2}$ in. and the maximum width $6\frac{1}{2}$ in. The circumference was $22\frac{3}{8}$ in.

The upper jaw had a full complement of sixteen teeth including the third molars (wisdom) teeth. On the left side fillings were present in the second bicuspid and the second and third molars, and on the right side in the first bicuspid and the second and third molars. The lower jaw contained thirteen teeth including the third molars (wisdom) teeth. The two incisors on the left side and the central incisor on the right side were missing and had fallen out after death. Fillings were present in the second and third molars on the left side and in the second and third molars on the right side.

X-ray films were taken of both clavicles, one fibula, the body of the sternum and two of the ribs, and these gave the following information:

(1) *Fibula*. The ends (epiphyses) had completely fused with the shaft of the bone, and this generally indicates an age over 20.

(2) *Clavicles*. The breast-bone end should be united to the main part of the bone at about the twenty-fifth year. In one of these bones this union had occurred, but in the other it had only partially occurred, suggesting an age a little below 25 years.

(3) *Sternum*. All portions should be united before the age of 25. They were united in this bone.

(4) *Ribs*. The articular surfaces had united to the body of the rib. This should occur by the age of 25 years.

There were large numbers of dead maggots on the surface of the bones, and masses of dead maggots in the orbits and the nasal cavities. It would appear that maggots had been the principal agents in removing the flesh from the bones.

From my examination of the pit and the skeleton, I have drawn the following conclusions:

(1) All the bones present could belong to one body.

(2) The body had been that of a man aged 23–25 years.

(3) The height of the deceased, as calculated from formula would have been between 5 ft. 8 in. and 5 ft. $9\frac{1}{2}$ in.

(4) The head was of fairly large size, and the hair mid to light brown.

(5) The bones did not show any evidence of injury.

(6) The body had been in a sitting posture with a padlocked chain around the neck fastened to the third step, a padlocked chain around the right wrist, and possibly also around some other part of the body, and a padlocked chain around both ankles.

(7) It would have been possible for the deceased to have put all these chains in position himself. Indeed, it would have been very difficult for a second party to have assisted in this process in the confined space of the pit.

(8) The cause of death on the evidence available must be speculative, but the following possibilities are mentioned:

(i) The man, having chained himself up, may have lost the keys (or not had keys) and been unable to free himself with the small hack-saw found. In this remote place he could easily have starved, and any calls for help would have been unheard.

(ii) It is possible that the concrete slab had been propped up with some small support, and that this had broken allowing the slab to fall and leaving the

PLATE XV

*The inspection pit with the concrete cover in position.*

PLATE XVI

*View taken with the camera looking down the inspection pit.*

PLATE XVII

*Close up of chains recovered from the pit. The top left includes portions of the third step down, and the chain enclosing the right radius and ulna includes a portion of the fourth (bottom) step: the bottom left is the chain which probably encircled the legs, found in the sludge at the bottom of the pit.*

man in darkness, and possibly with a poor supply of air and nothing in the way of fresh air.

(iii) In this type of "bondage" case it is quite possible for the deceased to have been accidentally strangled by the chain round the neck.

### IDENTIFICATION OF THE BODY

While these investigations were proceeding, evidence of a different character made further attempts at identification by medico-legal methods unnecessary.

(1) The pit had been inspected in 1954, and the body was not present at that time; this ruled out any form of incident having taken place while the camp was occupied.

(2) A person had been listed as missing, last seen in the Plymouth area, in the *Police Gazette* of 20th May, 1958, and an enquiry from Superintendent Langman to New Scotland Yard showed that the missing person was known as a "masochist and exhibitionist". This will be discussed later.

(3) In the sludge taken from the pit, Mr. W. Gliddon, staff biologist at the South Western Forensic Science Laboratory at Bristol, found an inscribed wrist watch, a pair of spectacles, a tobacco pouch, two padlock keys, and a broken hack-saw blade; seeing the name of the deceased engraved on the inside of the watch case was very helpful!

The father of the boy identified some of these articles and some of the portions of clothing. He knew that his son had been in Plymouth in March 1958, as a cheque made out to pay the son's hotel expenses on 22nd March had been returned to the father by the bank after his son's disappearance.

It was clear that this young man, aged 23, had left the hotel in Plymouth on 23rd March, 1958, and made his way to the disused camp at Lyneham; there he had found the inspection pit, descended it, and manacled himself to the steps by chains and padlocks. What happened after that is speculation, but the possibilities are mentioned in my report. In this type of case, of which the author has seen three examples, suicide is not usually contemplated, and death is accidental.

### PREVIOUS HISTORY

Evidence produced at the inquest filled in the background to these unusual events so clearly that it must be outlined. At the age of 14, the deceased boy had meningitis, but made a good recovery, although he had fits of depression from time to time. When he was 15, he was examined by a psychiatrist because he could not make the grade at a public school, and left to go to a school for backward children. After leaving school he served for three years as a private in the Army, and on leaving studied at a school of dramatic art. During this period, in April 1957, he had been found in London in a locked room in a basement, lying on his bed in his underpants, pinned to the frame of the bed by a cycle chain around his legs. He at first stated that he had met two men in a public house in Earl's Court and had taken them to his home; he later admitted that the chain and wire were his own property, and that he had allowed a friend to pin him to the bed.

There was a second incident in London in February 1958, just about a month before he finally disappeared from Plymouth. A constable was called to a derelict house where he heard a whistling noise coming from the basement. On entering he found this same young man, dressed in a windcheater, short trousers and gumboots, chained and padlocked to a metal banister by his wrists. It was necessary to call the fire brigade to release him. He first said that he had been chained to the banister by two men, but later admitted that he had chained and secured himself by means of a self-locking padlock, and that he had carried out this experiment in an attempt to gain sexual satisfaction.

It seems clear that the events in the pit at Lyneham were a further, more elaborate form of this perversion known as "bondage"; it is part of the pattern of this perversion that increasingly elaborate forms are required to give the necessary satisfaction, and it is obvious that a fatal accident, especially strangulation, can easily occur. Self-induced partial asphyxia is indeed sometimes part of the cult.

The deceased saw a psychiatrist, at his father's request, on the day before his disappearance. The psychiatrist formed an opinion that the young man had an inadequate personality and an inability to adjust himself to his circumstances; the condition was complicated by one of the less common types of sexual perversion.

The Coroner returned a verdict of accidental death, either from asphyxiation or strangulation, it being impossible to determine the exact mode of death.

I wish to thank G. G. Pearse, Esq., at that time H.M. Coroner for the Tavistock District of Devon for permission to publish this report.

## Figures and Tables

**Figure f1:**
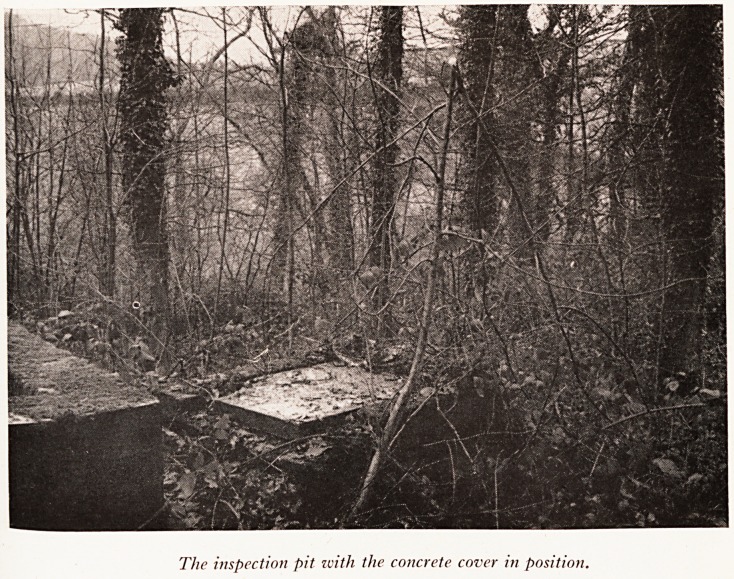


**Figure f2:**
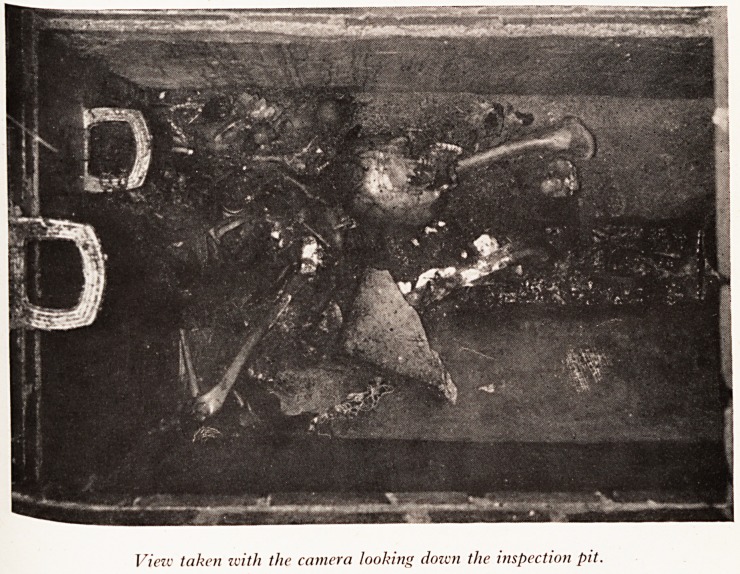


**Figure f3:**